# Screening Endophytic Fungi of Australian Indigenous Plants for Biosynthesis of Silver Nanoparticles and Evaluation of Their Antibacterial Properties

**DOI:** 10.3390/molecules31193505

**Published:** 2026-10-01

**Authors:** Hanadi Sawalha, Simon E. Moulton, Bita Zaferanloo

**Affiliations:** 1 Department of Chemistry and Biotechnology, School of Science, Computing and Emerging Technologies, Swinburne University of Technology, Melbourne, VIC 3122, Australia; hsawalha@swin.edu.au; 2 Department of Engineering Technologies, School of Engineering, Swinburne University of Technology, Melbourne, VIC 3122, Australia; smoulton@swin.edu.au; 3 The Aikenhead Centre for Medical Discovery, St Vincent’s Hospital, Melbourne, VIC 3065, Australia; 4 Iverson Health Innovation Research Institute, Swinburne University of Technology, Melbourne, VIC 3122, Australia

**Keywords:** endophyte, AgNPs, biosynthesis, bacterial resistance, *Pseudomonas aeruginosa*, *Staphylococcus aureus*

## Abstract

The increasing emergence of bacterial resistance to conventional antimicrobial agents underscores the urgent need for alternative therapeutics. In this context, the present study investigated the antibacterial potential of silver nanoparticles (AgNPs) biosynthesised from extracellular extracts of four endophytic fungal species isolated from *Eucalyptus globulus* and *Eucalyptus citriodora*. The AgNPs were comprehensively characterised using UV–visible spectroscopy, Fourier-transform infrared spectroscopy (FTIR), transmission electron microscopy (TEM), and zeta potential analysis. UV–vis spectra confirmed nanoparticle formation through characteristic absorption peaks between 400 and 500 nm. TEM imaging revealed predominantly spherical nanoparticles, with size ranges of 52–98 nm for EG 5 AgNPs, 86–197 nm for EG 6 AgNPs, 56–171 nm for EG 8 AgNPs, and 50–130 nm for EC 2 AgNPs. High negative zeta potential values (−19.3 to −31.7 mV) indicated moderate colloidal stability. FTIR analysis identified functional groups associated with the reduction and stabilisation of AgNPs. All AgNPs samples exhibited notable antibacterial activity against both *Staphylococcus aureus* and *Pseudomonas aeruginosa*, and growth–kill curve analysis demonstrated a marked reduction in bacterial proliferation over 24 h. Collectively, these findings highlight the potential of endophytic fungus-derived AgNPs as effective antibacterial agents.

## 1. Introduction

Antibiotic resistance, defined as the failure of antimicrobial agents to inhibit bacterial growth [1], continues to rise due to the widespread misuse and overuse of antibiotics [2]. This escalation has contributed to increased morbidity and mortality associated with infectious diseases worldwide [3], placing substantial strain on healthcare systems. As resistance intensifies, higher antibiotic doses are often required to achieve therapeutic effects, frequently leading to increased toxicity, disruption of the host microbiota, and diminished patient compliance [4]. The emergence of multidrug-resistant bacterial strains, particularly among both Gram-positive and Gram-negative pathogens, has further limited available treatment options, underscoring the urgency for alternative antimicrobial strategies that are both effective and sustainable [5].

Nanotechnology has emerged as a promising avenue to address these challenges, offering innovative approaches to antimicrobial therapy [6]. Nanoparticles (NPs) exhibit unique physicochemical properties, including high surface-area-to-volume ratios and size-dependent biological interactions, which enable enhanced antibacterial activity compared with conventional agents [7,8]. Importantly, NPs exert antimicrobial effects through multiple mechanisms, such as membrane disruption, oxidative stress induction, inhibition of protein synthesis, and interference with DNA replication, making it more difficult for bacteria to develop resistance [9]. Among metallic NPs, silver nanoparticles (AgNPs) are particularly notable due to their broad-spectrum antibacterial activity, long-term effectiveness, and relatively low propensity for inducing resistance [6,10].

Growing environmental and health concerns associated with conventional chemical and physical synthesis methods, such as the use of toxic reductants, high energy demands, and the generation of hazardous by-products, have driven increased interest in green synthesis approaches [11]. Biosynthesis using biological systems, including bacteria, fungi, yeasts, and plant extracts, provides an environmentally friendly alternative that minimises toxic waste, reduces cost, and improves biocompatibility [12].

Among these biological systems, fungi have attracted particular attention for their ability to secrete large quantities of extracellular enzymes, proteins, and secondary metabolites that facilitate efficient metal-ion reduction and nanoparticle stabilisation [13,14]. Endophytic fungi, microorganisms that reside asymptomatically within plant tissues, offer additional advantages as NP producers [15]. Endophytic fungi contribute significantly to plant growth and development by influencing metabolic processes and facilitating the uptake of nutrients and other beneficial compounds from the environment, including soil, water, and organic matter [16]. Thus, their metabolic diversity, shaped by intimate plant–fungus interactions, enables the production of a wide range of bioactive compounds with reducing and capping capabilities [13]. Moreover, endophytic fungi grow rapidly, can be maintained on inexpensive substrates, and allow extracellular NP synthesis, simplifying downstream processing [17,18,19]. These features position endophytic fungi as efficient and scalable “nano-factories” capable of producing stable and biologically active AgNPs [20].

Despite this promise, biosynthesis of AgNPs using endophytic fungal extracts remains less explored than plant- or bacteria-mediated approaches. Existing studies have focused mainly on bacterial supernatants [21] and plant extracts [22], while the biochemical complexity of fungal extracts continues to pose challenges for reproducibility and standardisation [23]. Variations in fungal species, growth conditions, developmental stages, and extraction protocols can significantly influence extract composition, affecting AgNP growth, size distribution, and stability [24].

Given these gaps, the present study aimed to synthesise AgNPs using extracellular extracts from four endophytic fungal isolates, characterise the resulting AgNPs using visual assessment, UV–visible spectroscopy, zeta potential analysis, FTIR, and TEM, and evaluate their antibacterial activity against *Staphylococcus aureus* (*S. aureus*) and *Pseudomonas aeruginosa* (*P. aeruginosa*). Therefore, the current study indicates that four *Alternaria* isolates, derived from Australian endogenous plants, differed in their nanoparticle synthesis performance and antibacterial efficacy, suggesting that isolate-specific metabolic traits play a critical role in nanoparticle formation. Variations in extracellular proteins, enzymes, and secondary metabolites among the endophytic fungal isolates may influence Ag^+^ reduction and antibacterial activity [25]. Therefore, the comparative approach adopted in this study provides valuable insights beyond simply confirming fungal-mediated AgNP synthesis. The findings highlight that selecting fungal isolates is an important factor influencing nanoparticle quality and antibacterial effectiveness.

## 2. Results and Discussion

### 2.1. Identification of Endophytic Fungal Isolates and Phylogenetic Analysis

Each purified isolate was assigned a unique code based on the host plant and the order in which it was isolated. Isolates obtained from *Eucalyptus globulus* were labelled “EG,” followed by a numerical identifier (e.g., EG5, EG6, EG8), whereas isolates from *Eucalyptus citriodora* were labelled “EC,” such as EC2. These codes were used consistently throughout the study to track the origin of each fungal extract and its corresponding biosynthesised AgNPs.

**Figure 1:**
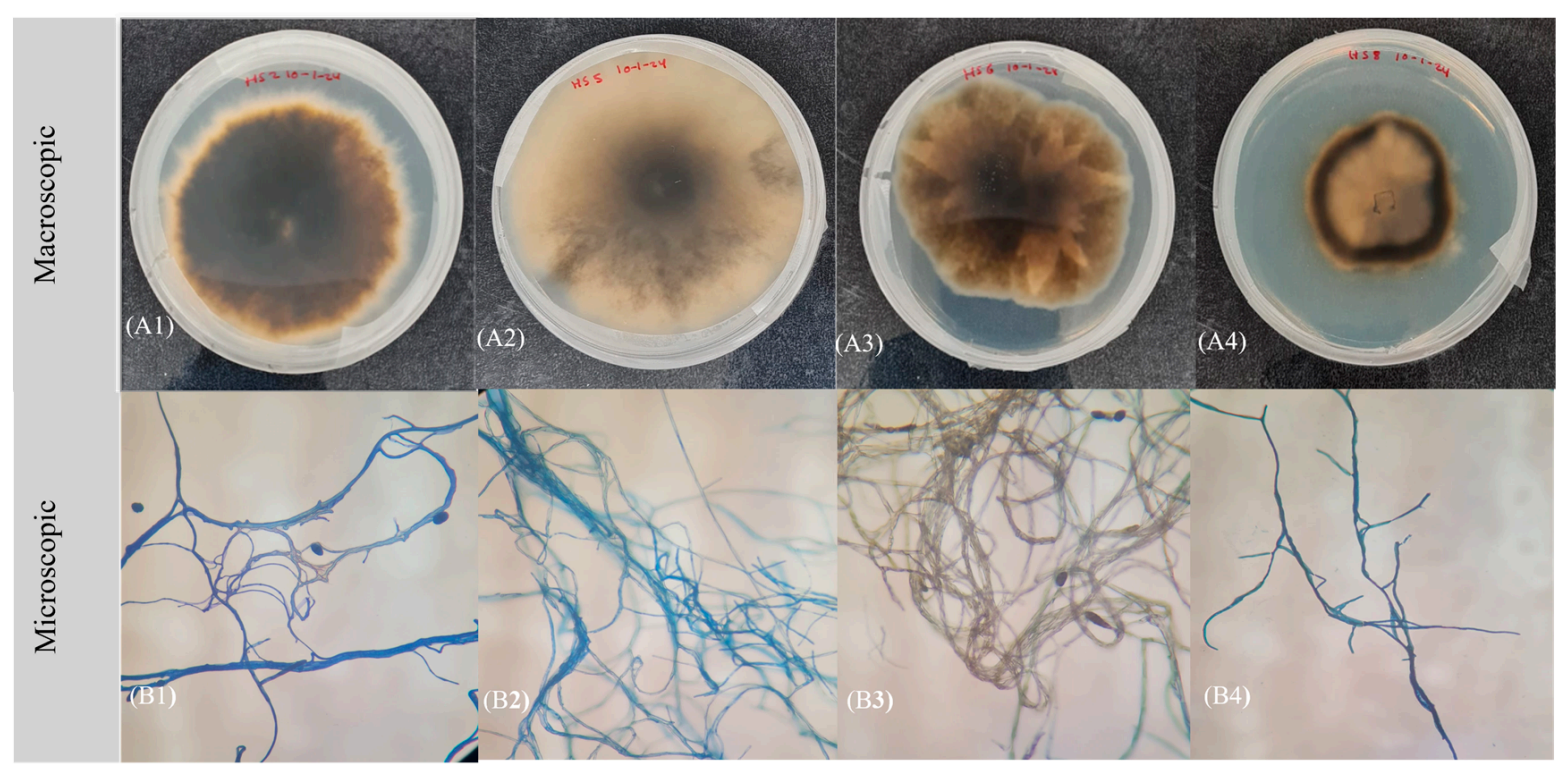
Macroscopic observations of endophytic fungal isolates (**A1**–**A4**), EG5, EG6, EG8, and EC2, respectively. Microscopic observations at 40× magnification reveal the hyphal structure of endophytic fungi (**B1**–**B4**), EG5, EG6, EG8, and EC2, respectively.

Morphological and molecular identification was used to identify the endophytic fungal isolates EG5, EG6, EG8, and EC2. Depending on the colony characteristics, the mycelial colour of EG5 was initially white and became grey when mature. EG 6 was chocolate brown, EG 6 was pale brown with dark-brown edges, and EC2 exhibited dark- and pale-brown edges, as depicted in Figure 1. 

Molecular identification based on amplification of the ITS region yielded PCR products of approximately 500 bp for all isolates. BLASTn analysis of the sequenced ITS fragments showed 100% identity with *Alternaria tenuissima*, *Alternaria alternata*, *Alternaria brassicae*, and *Alternaria destruens* for EG5, EG6, EG8, and EC2, respectively. The sequences were deposited in NCBI GenBank under accession numbers PZ134488, PZ134489, PZ134490, and PZ134491 for EG5, EG6, EG8, and EC2, respectively. Multiple sequence alignment was performed using ClustalW with default parameters, and phylogenetic relationships were inferred using the maximum likelihood method, as shown in Figure S1. The robustness of the tree topology was evaluated through bootstrap analysis with 1000 replications, confirming strong support for the clustering of the isolates within their respective Alternaria lineages.

### 2.2. Biosynthesis of AgNPs by Endophytic Fungi

**Figure 2:**
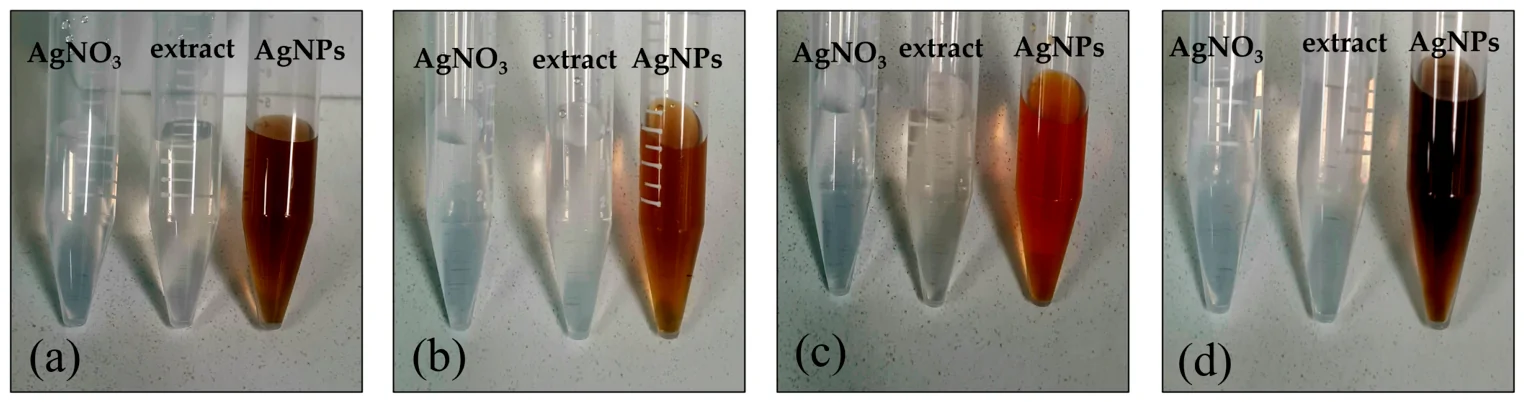
AgNO_3_ solution, fungal extract, and colour change indicating the biosynthesis of AgNPs from (**a**) EG5, (**b**) EG6, (**c**) EG8, and (**d**) EC2.

Each isolated extract was evaluated for its ability to synthesise AgNPs following the experimental methods previously described. Before mixing with the reagents, all the extract solutions were clear, and upon mixing, the combined solution changed colour to brown/dark brown over a period of 72 h, as shown in Figure 2, associated with the formation of AgNPs [26].

**Figure 3:**
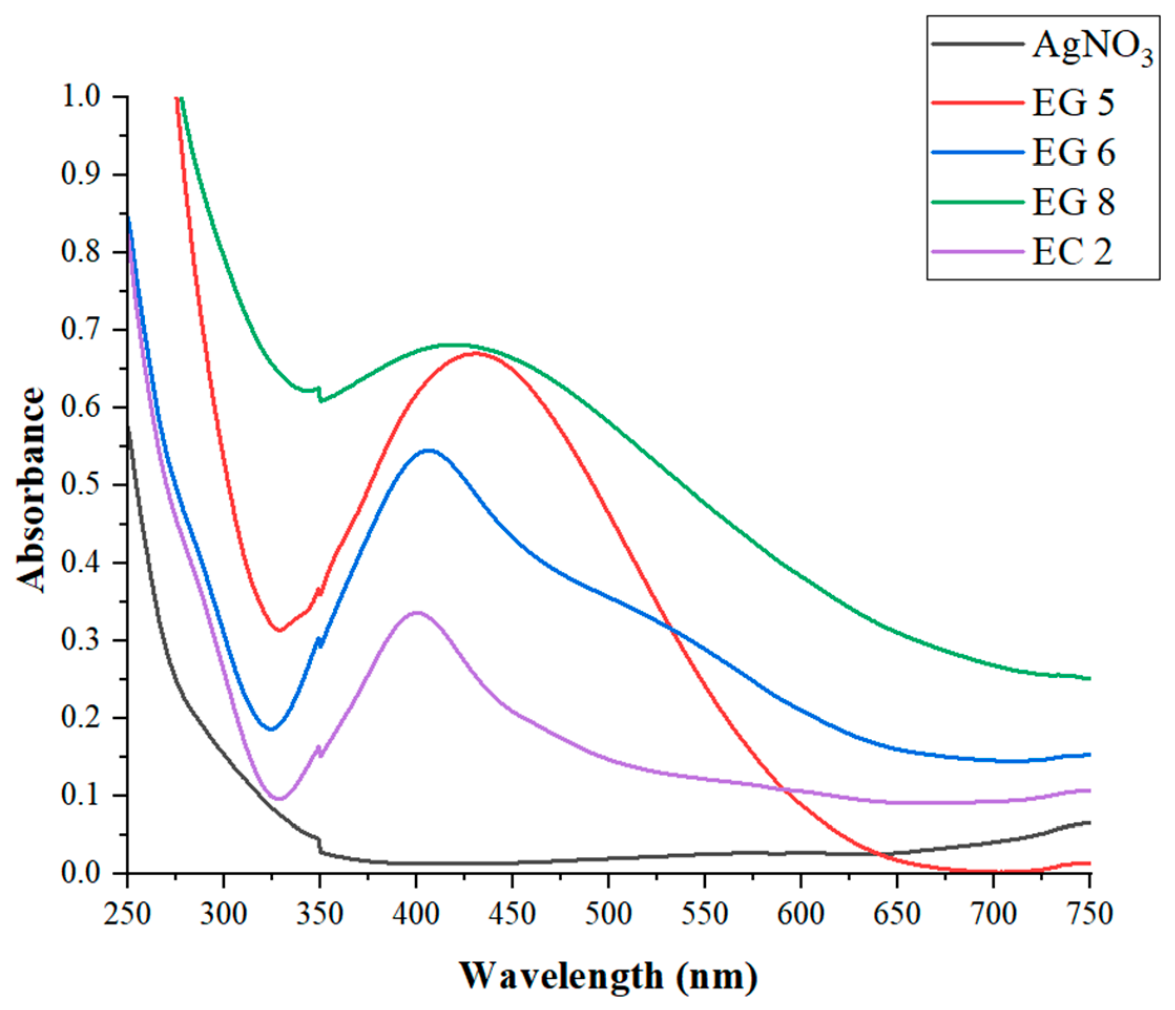
UV–Vis spectra of biosynthesised AgNPs after 72 h of reaction. The spectra display a characteristic SPR peak between 400 and 500 nm, confirming AgNP formation. AgNO_3_ solution was included as the control.

The colour shift resulted from a reduction of Ag^+^ to Ag^0^ and AgNPs in the solution [27]. Dadayya et al. [28] confirmed the reduction of Ag^+^ to AgNPs by the colour change of the extracellular extract of Alternaria carthami to dark brown. The reduction of Ag^+^ to AgNPs is indicated by the colour change of the extracellular extract of *Alternaria alternata* from pale yellow to dark brown [29]. The colour shift may result from the surface plasmon resonance (SPR), a phenomenon in which electrons from the nanoparticle’s positive and negative charges oscillate and interact with each other [30]. These findings were validated by UV–visible spectral analysis, showing an absorption band between 400 and 500 nm, as illustrated in Figure 3 [31]. The bio-reduction of silver occurs due to the presence of a reducing agent in the extract, which has been shown to convert Ag^+^ in AgNO_3_ solution to Ag0 at the nanoscale, as represented in Equation (1) [32].

Ag^+^ + e^−^ → Ag^0^ (1)

### 2.3. Characterisation of Biosynthesised AgNPs

#### 2.3.1. UV–Visible Analysis

UV–visible absorption spectroscopy is a direct and effective technique for verifying nanoparticle synthesis [33]. The UV–vis spectra in Figure 3 show absorption maxima (λ max) between 400 and 500 nm. These SPR peaks of all AgNPs samples provide compelling evidence for the presence of AgNPs [34]. The differences detected in the UV–Vis spectra among several extracts suggest that each extract uniquely affects the formation and stability of AgNPs, attributable to differences in their phytochemical composition [35]. The width of the SPR peak represents the NPs size distribution, with a narrow peak indicating uniform (monodisperse) NPs, while a broader peak denotes polydispersity and likely aggregation [36]. Optimal AgNPs synthesis typically requires an SPR peak around 420 nm exhibiting a high intensity and a narrow bandwidth, indicating the presence of small, stable, and evenly distributed NPs with high colloidal stability [37].

The SPR peak of EG5 AgNPs occurred at 431 nm, characterised by a narrow width and higher absorbance, indicating well-dispersed, monodisperse AgNPs of uniform size. In contrast, the SPR peak of EG6 AgNPs occurred at 408 nm, which has a broader peak with higher absorbance, indicating a polydisperse size distribution, likely caused by aggregation or the presence of NPs spanning a wider range of sizes. The SPR peak of EG8 AgNPs, while narrow, displays reduced absorbance, suggesting the presence of smaller, monodisperse NPs than in EG5 AgNPs. Likewise, the SPR peak of EC2 AgNPs is narrow with similarly low absorbance, indicating small NPs, with a possible tendency toward slight aggregation.

The broad peak at ~550 nm observed for EG 6 is indicative of larger particle sizes, increased polydispersity, and partial aggregation, as SPR band broadening and red-shifting are commonly associated with interparticle interactions. This is consistent with the corresponding DLS data for EG 6, which showed a larger average hydrodynamic diameter and a higher polydispersity index than the other samples. These additional UV–Vis features arise from variations in fungal extract composition and NP size distribution, highlighting the role of fungal-derived biomolecules in influencing AgNP sizes, stability, and optical properties.

#### 2.3.2. Dynamic Light Scattering Analysis

The size, zeta potential, and polydispersity index (PDI) of the four biosynthesised AgNP samples were analysed using a Zetasizer, with the results summarised in Table 1. Dynamic light scattering (DLS) measurements in Figure S2 show average hydrodynamic diameters, obtained directly from the instrument’s intensity-weighted numerical output, of approximately 80 nm, 158 nm, 121 nm, and 104 nm for EG 5, EG 6, EG 8, and EC 2, respectively.

Several DLS traces exhibited broadened distributions and secondary peaks at larger sizes, indicating the presence of more than one particle population within the samples [38]. The dominant peaks correspond to the primary AgNP population, whereas the secondary peaks are likely attributable to larger particles or NP aggregates, which scatter light more intensely and therefore appear prominently in intensity-based DLS measurements [39]. The occurrence of multiple peaks thus reflects polydispersity and partial aggregation, rather than the dominance of large particles in the samples [40].

This interpretation is supported by the PDI values obtained previously in this study for each AgNP formulation. The PDI of EG 5 AgNPs was 0.19, indicating a highly monodisperse and uniformly dispersed nanoparticle population. In contrast, EG 8 AgNPs displayed a moderate level of polydispersity (PDI = 0.36), while EG 6 and EC 2 AgNPs exhibited higher polydispersity, with PDI values of 0.41 and 0.52, respectively. Higher PDI values correlate with broader DLS size distributions and the presence of secondary peaks, consistent with increased heterogeneity and a tendency toward aggregation. As reported by [41,42], a high scattering intensity combined with a low PDI reflects well-dispersed AgNPs. In contrast, high intensity with an elevated PDI indicates aggregation and the presence of larger particle populations.

The zeta potential values of the AgNPs were −19.3 mV (EG 5), −31.7 mV (EG 6), −18.1 mV (EG 8), and −21.9 mV (EC 2). The negative surface charges indicate electrostatic repulsion between particles, which contributes to colloidal stability by reducing nanoparticle agglomeration. Samples with more negative zeta potential values, particularly EG 6, are expected to show improved electrostatic stabilisation [43]; however, the observed polydispersity suggests that variations in surface capping efficiency and biomolecular composition of the fungal extracts also play a key role in governing aggregation behaviour and size heterogeneity [44]. Overall, the combined DLS, PDI, and zeta potential data demonstrate that while the biosynthesised AgNPs are predominantly nanoscale, their dispersity and aggregation state vary among samples due to differences in synthesis conditions and extract composition.

#### 2.3.3. FTIR Analysis

**Figure 4:**
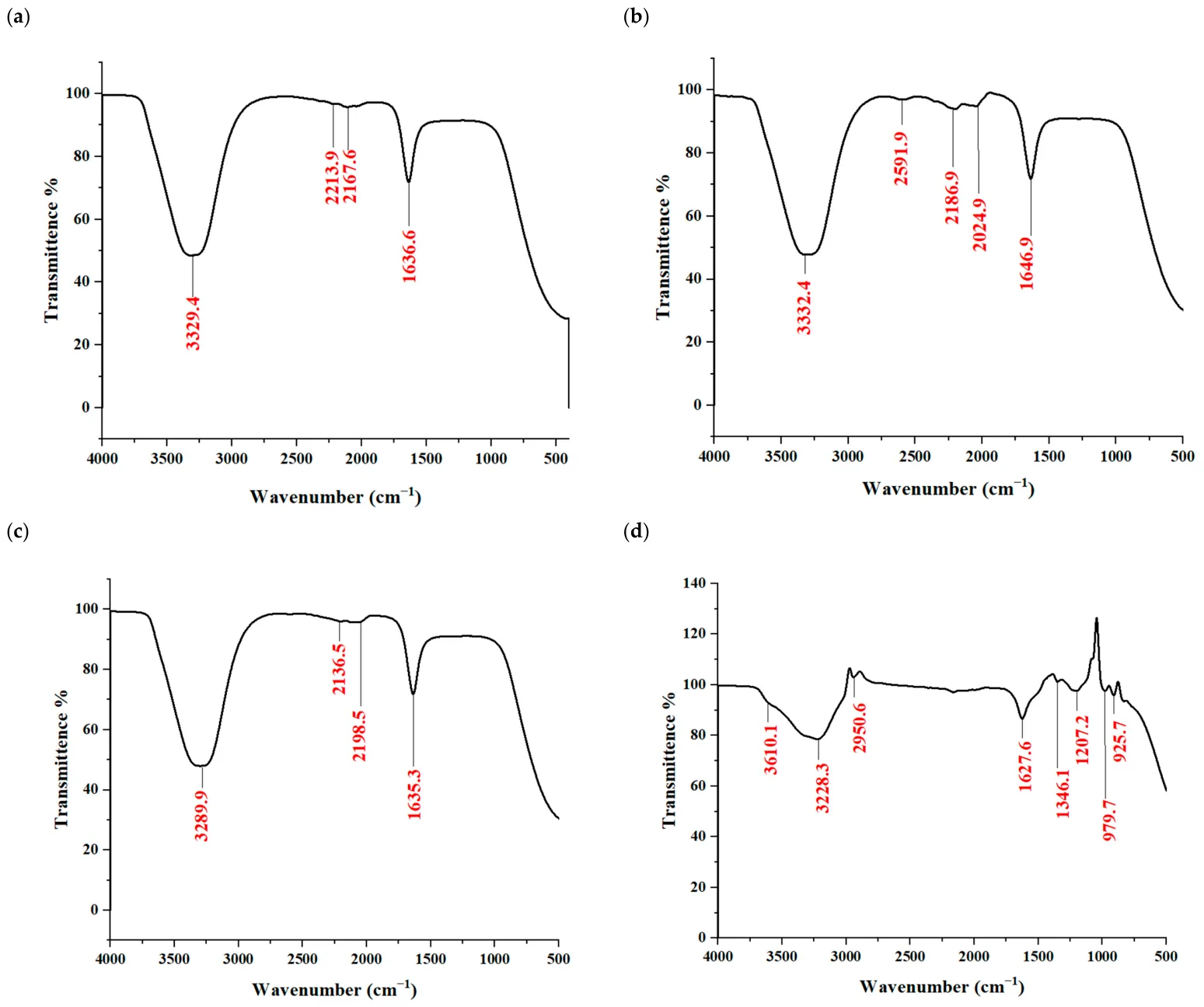
FTIR analysis of AgNPs synthesised from (**a**) EG5, (**b**) EG6, (**c**) EG8, and (**d**) EC2, showing the functional groups associated with AgNP formation.

The vibration and rotation modes of functional groups present in the biomolecules attached to the surface of the AgNPs were detected using FTIR and were used to identify the various functional groups present on the surface of biosynthesised AgNPs, as shown in Figure 4. The FTIR spectrum of EG 5 AgNPs showed peaks at 3329.4, 2213.9, 2167.6, and 1636.6 cm^−1^, representing amine N–H stretching, C≡N stretching of nitrile, C≡N stretching, and C=O stretching and bending of secondary amide from proteins, respectively. EG 6 AgNPs showed peaks at 3332.4, 2591.9, 2186.9, 2024.9, and 1646.9 cm^−1^. The peaks correspond to N–H stretching, S–H stretching of thiol, C≡C of alkynes, aromatic biomolecules, and C=O stretching of primary amide from proteins, respectively. The analysed peaks of EG8 AgNPs were observed at 3289.9, 2136.5, 2198.5, and 1635.3 cm^−1^. The peaks indicate O–H stretching from hydroxyl groups, C≡N stretching of nitriles, C≡C stretching of alkynes, and C=O stretching of amides. EC 2 AgNPs showed peaks at 3610.1, 3228.3, 2950.6, 1627.6, 1346.1, 1207.2, 979.7, and 925.7 cm^−1^. The peaks suggest O–H stretching, N–H stretching, C–H vibrations of alkanes, C=O stretching, C–N stretching of amines, C–O stretching of phenols, and =C–H bending of alkenes. The extracellular proteins in the fungal extract are the likely source for the reduction of Ag^+^ to Ag 0, with these proteins also stabilising the AgNPs by binding to them and acting as capping agents [45].

#### 2.3.4. TEM Analysis

**Figure 5:**
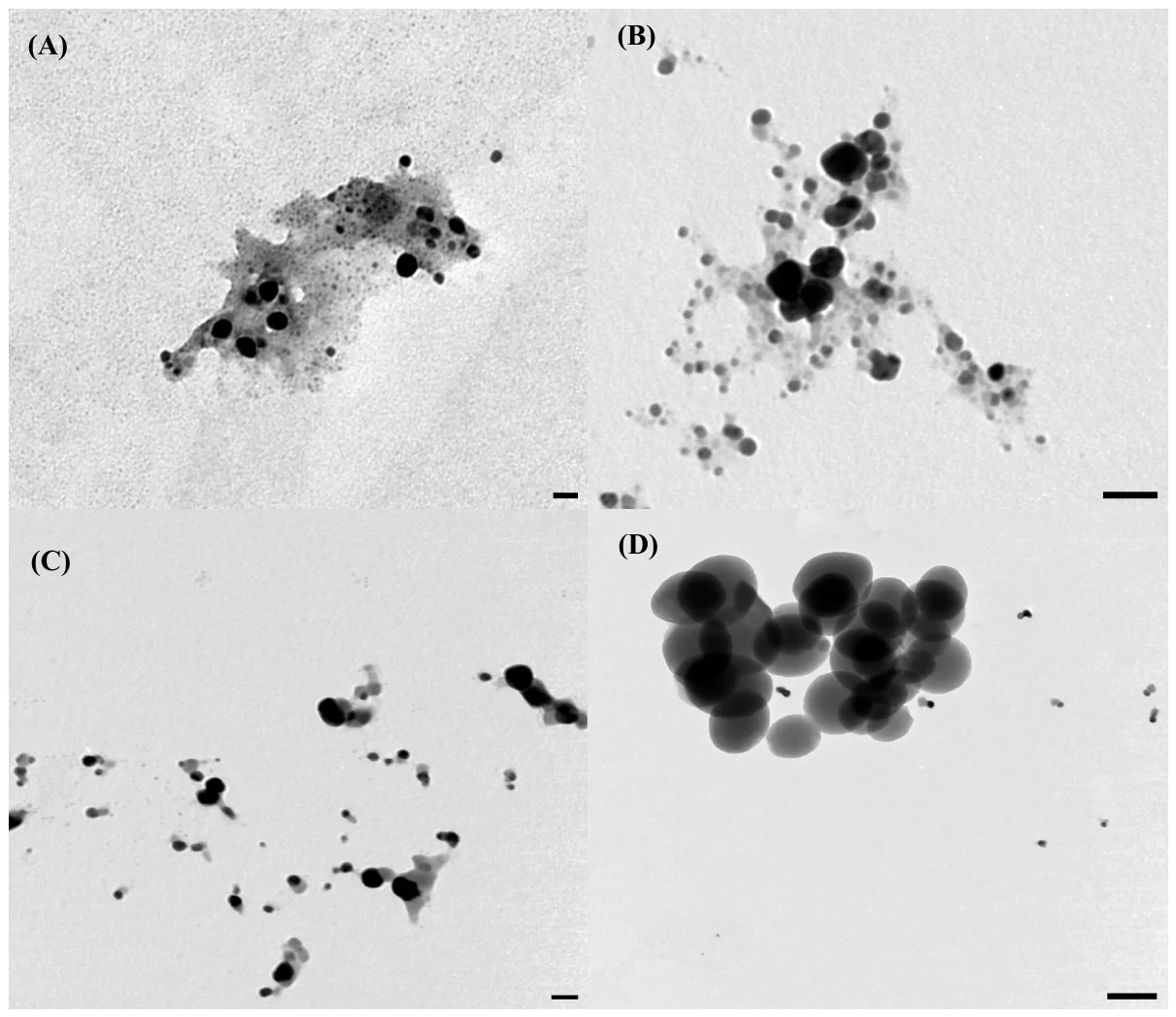
TEM images of AgNPs synthesised by (**A**) EG5, (**B**) EG6, (**C**) EG8, and (**D**) EC2, showing AgNP morphology and distribution. Scale bar = 100 nm.

TEM analysis was conducted to provide quantitative measures of the size and morphology of the biosynthesised AgNPs [35]. Based on Figure 5, TEM micrographs indicate that AgNPs were approximately spherical in shape. The average size of EG5 AgNPs ranged from 52 to 98 nm, whilst the size range for EG6 AgNPs was from 86 to 197 nm, that of EG8 AgNPs was from 56 to 171 nm, and that of EC2 AgNPs was from 50 to 130 nm. Variations in AgNP sizes were observed among the four samples. The results indicate that the biosynthesised AgNPs exhibited average diameters of 78 ± 8 nm, 153 ± 19 nm, 119 ± 17 nm, and 94 ± 11 nm for EG5, EG6, EG8, and EC2, respectively, demonstrating differences in nanoparticle characteristics between the fungal isolates.

Morphological characteristics depend on the microorganisms and on the biosynthesis process parameters, such as incubation time, temperature, and pH of the reaction medium. In this study, all synthesis conditions were kept constant, with the only variable being the extract component. Therefore, the differences in size and morphology observed in the TEMs are attributed to the different extracts of endophytic fungal species used [46].

The slight difference between the size range obtained from TEM and the zeta sizer is attributed to the fact that in TEM, the sample is subjected to high vacuum while on the grid, resulting in the collapse of the polymer corona into a desiccated layer, which leads to particles appearing smaller than the hydrodynamic sizes recorded by the zeta sizer [47]. DLS measures the hydrodynamic diameter of NPs in suspension, which includes the particle core together with surface-bound biomolecules, solvent layers, and any soft aggregation, whereas TEM provides the approximately actual core size of dried, individual NPs [48]. Consequently, DLS values are typically larger than TEM sizes, particularly for biosynthesised nanoparticles coated with fungal-derived organic capping agents [49]. Despite these differences, both techniques show a consistent trend, with EG 5 producing the smallest AgNPs and EG 6 yielding the largest and most polydisperse particles. Moreover, TEM images provided a clear visual analysis of NPs’ size, shape, and dispersity. Smaller NP size and lower PDI were associated with well-dispersed and uniformly shaped AgNPs, while larger NPs with higher PDI showed variation in size and shape, as well as obvious clustering or aggregation.

#### 2.3.5. XPS Analysis

**Figure 6:**
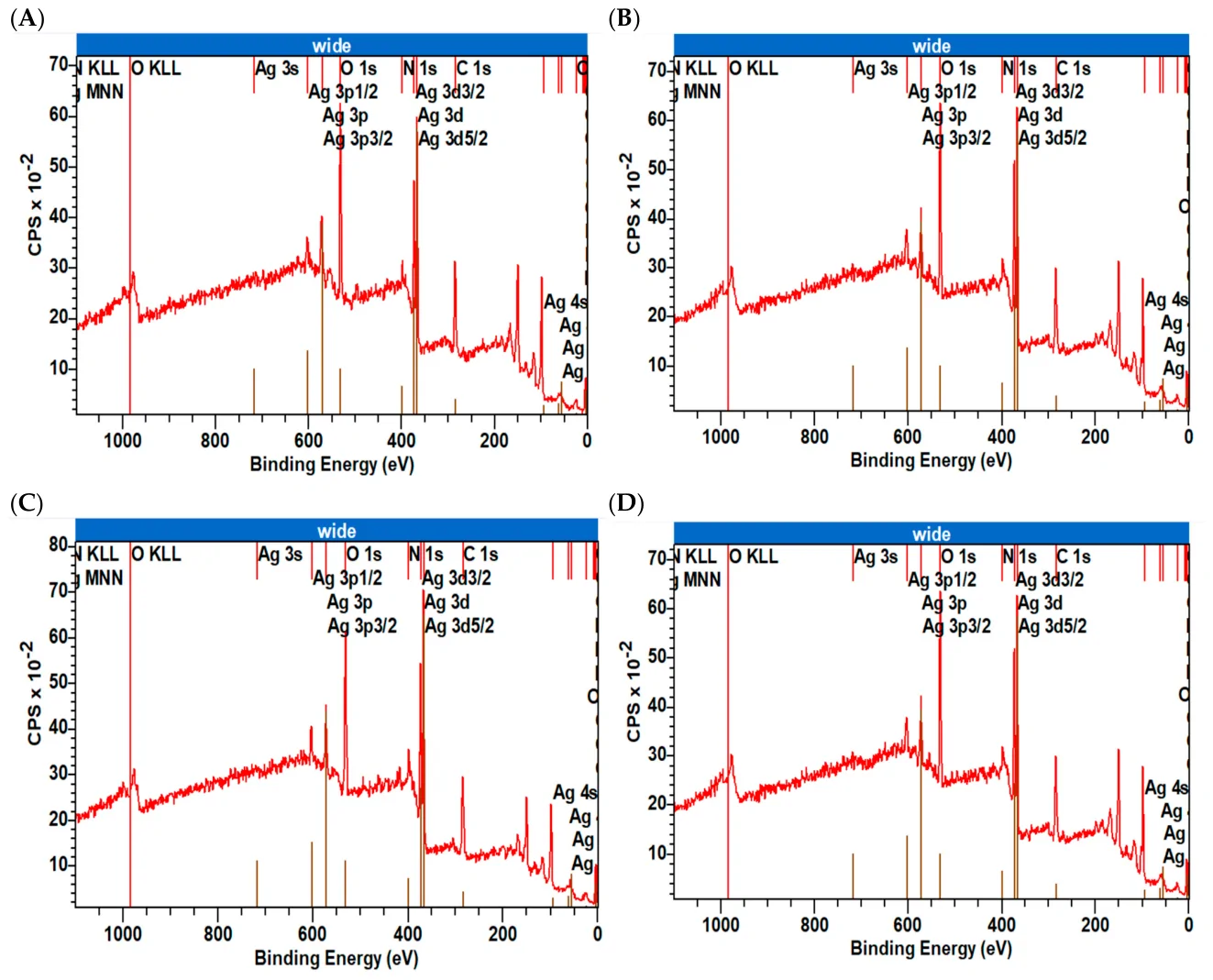
XPS analysis of AgNPs biosynthesised using endophytic fungal extract. The survey spectrum demonstrates the presence of Ag, C, O, and N elements on the nanoparticle surface for samples (**A**) EG5, (**B**) EG6, (**C**) EG8, and (**D**) EC2.

The chemical composition and oxidation state of the optimised AgNPs were investigated using X-ray photoelectron spectroscopy (XPS). The XPS survey spectrum revealed the presence of Ag, C, O, and N elements, confirming the surface elemental composition of the nanoparticles, as shown in Figure 6.

Across all four samples, the detected signals were characteristic of AgNPs. Prominent peaks corresponding to Ag 3d_5_/_2_ and Ag 3d_3_/_2_ were observed at approximately 368 eV and 374 eV, respectively. Additional silver-related peaks, including Ag 3p at approximately 573–604 eV, Ag 3s around 719 eV, and low-binding-energy features associated with Ag 4s/4p, were also identified. The consistent presence of these characteristic silver peaks across all samples confirms the successful incorporation of silver and verifies the formation of AgNPs in each formulation [50].

The spectra also contain C 1s around 285 eV, O 1s around 530–533 eV, and N 1s around 399–401 eV. These signals suggest that organic molecules are associated with the nanoparticle surfaces [51]. Carbon, oxygen and nitrogen can originate from proteins, amino acids, polysaccharides or other endophytic fungal metabolites involved in the reduction of the nanoparticles [52].

The overall elemental composition was consistent across all four samples, with Ag, C, O, and N detected in each case. However, noticeable variations were observed in the relative intensities of several peaks, particularly those corresponding to O 1s, N 1s, Ag 3d, and other silver-related signals. These differences may reflect variations in the quantity or composition of biomolecules adsorbed onto the AgNP surface [53]. Such variations in surface chemistry could influence the physicochemical properties and biological activity of the biosynthesised AgNPs [54].

### 2.4. Antibacterial Susceptibility Test

#### 2.4.1. MIC and MBC Measurements

After 24 h of incubation under aerobic conditions at 37 °C, turbidity was observed in the test tubes containing EG5 AgNPs (60–80 µg/mL) and EG6, EG8 and EC2 AgNPs (60–100 µg/mL) against *S. aureus*, as shown in Table 2. Turbidity was also detected in tubes containing EG5 AgNPs (60–400 µg/mL) and EG6, EG8 and EC2 AgNPs (60–800 µg/mL) against *P. aeruginosa*, as presented in Table 3. Based on these observations, the MIC values for *S. aureus* were determined to be 100 µg/mL for EG5 AgNPs and 400 µg/mL for EG6 AgNPs, EG8 AgNPs, and EC2 AgNPs. For *P. aeruginosa*, the MIC was 800 µg/mL for EG5 AgNPs and 1000 µg/mL for EG6, EG8, and EC2 AgNPs.

**Figure 7:**
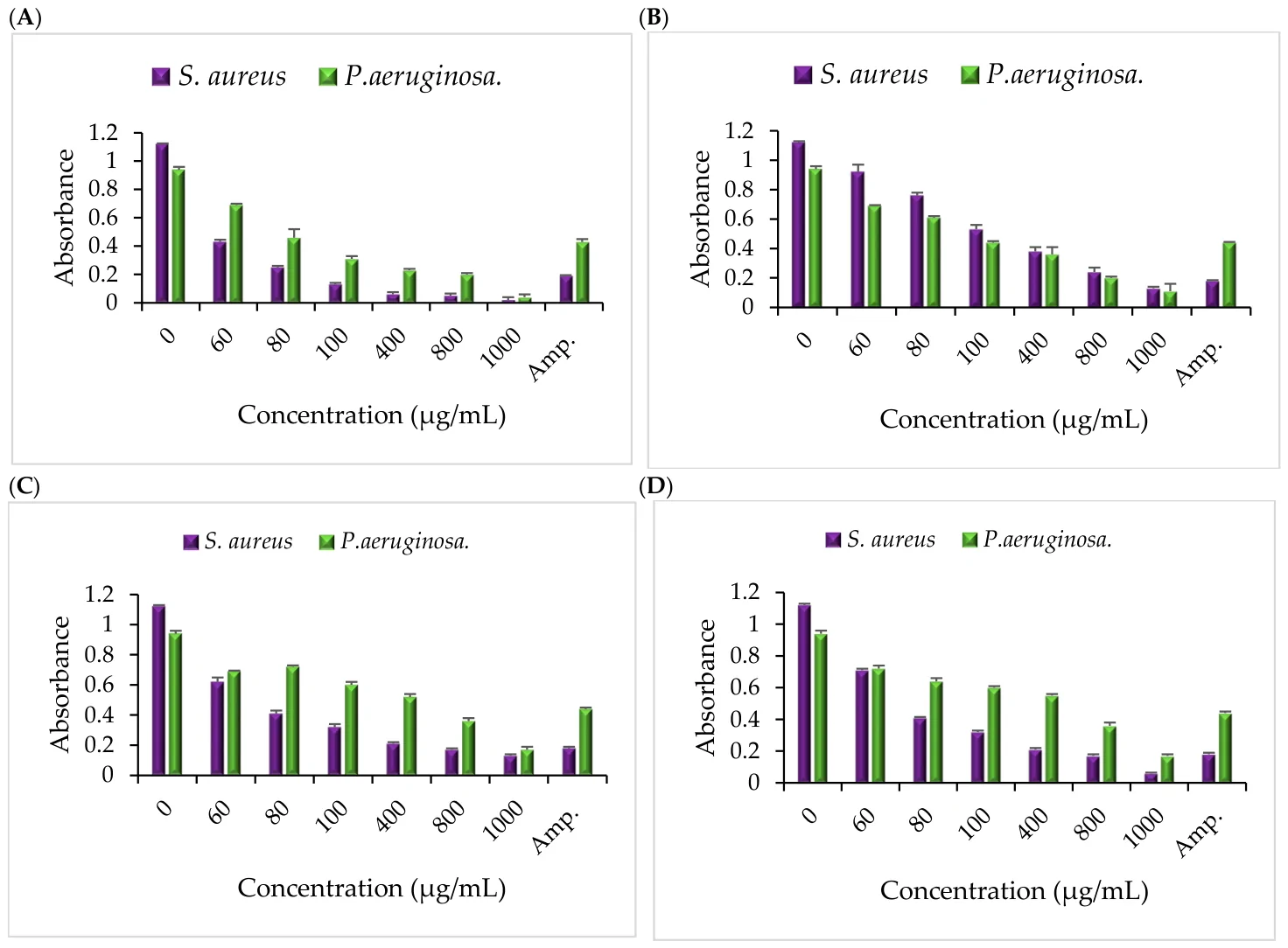
MIC concentrations of AgNPs from (**A**) EG5, (**B**) EG6, (**C**) EG8, and (**D**) EC2, against *Staphylococcus aureus* and *Pseudomonas aeruginosa*. The absorbance represents the OD_600nm_-corrected readings, indicating the growth of *S. aureus* and *P. aeruginosa* after 24 h of incubation; bacterial growth at 0 µg/mL was used as the negative control, and ampicillin (Amp.) 20 µg/mL was used as the positive control in this study. The results are presented as mean ± SD.

The findings presented in Figure 7 are consistent with the visual observations reported in Table 2 and Table 3. The MIC screening of AgNPs produced by individual isolates (EG5, EG6, EG8, and EC2) against *S. aureus* and *P. aeruginosa* was assessed by OD_600nm_ measurements after 24 h of incubation. For AgNPs from EG5, a concentration-dependent reduction in absorbance was observed for *S. aureus*, with marked growth inhibition evident from 100 µg/mL onward, whereas against *P. aeruginosa*, they displayed a more gradual decrease in absorbance and retained measurable growth at higher concentrations from 800 µg/mL µg/mL onward. In the case of EG6, both *S. aureus* and *P. aeruginosa* showed progressive reductions in absorbance with increasing AgNP concentrations from 400 µg/mL and 100 µg/mL onward, respectively. For isolate EG8, inhibition of *S. aureus* was evident across the tested concentration range, while *P. aeruginosa* demonstrated comparatively higher tolerance, with residual growth persisting at intermediate concentrations. AgNPs produced by EC2 also resulted in concentration-dependent growth inhibition of both bacterial species; however, *S. aureus* again showed a more pronounced reduction in absorbance relative to *P. aeruginosa*. Across all isolates, ampicillin (20 µg/mL) was used as the positive control and resulted in reduced absorbance for both bacterial strains, while the untreated control (0 µg/mL) showed maximum growth.

EG5 AgNPs demonstrated enhanced antibacterial activity. This activity may be associated with their smaller particle size compared with the other biosynthesised AgNPs [55]. Nanoparticles with smaller dimensions possess a higher surface-area-to-volume ratio, providing more active sites for interaction with bacterial cells [56]. This increased surface reactivity can enhance membrane attachment, cellular penetration, and silver ion release, all of which contribute to bacterial growth inhibition [57]. Since EG5 AgNPs exhibited the smallest size range among the biosynthesised nanoparticles, their greater bioavailability and interaction with bacterial cells likely explain their enhanced antibacterial efficacy.

Previous studies have revealed that biosynthesised AgNPs showed high antibacterial activity. Naik at al. [58] reported that the MIC of biosynthesised AgNPs against *S. aureus* is 1000 µg/mL. Hosnedlova et al. [59] found that the MIC of biosynthesised AgNPs against *S. aureus* is 25 µg/mL. Another study found that the MIC of biosynthesised AgNPs is 62.5 µg/mL against *S. aureus*, and about 200 µg/mL of biosynthesised AgNPs was found to be the MIC value against *P. aeruginosa* [60]. Differences between the present findings and previously reported results may stem from variations in NPS physicochemical properties, including size, morphology, and aggregation state, as well as phytochemical compounds in endophytic fungal extract composition when green synthesis is employed [61]. Additional factors such as differences in bacterial strains, inoculum density, growth media, incubation conditions, and NP stability during storage, and methodological inconsistencies across laboratories, can further contribute to discrepancies [62].

The MBC assay is used alongside MIC testing to evaluate the ability of an antibacterial compound to inhibit bacterial growth [63]. The absence of colony formation on MHA plates indicated complete bacterial killing by the biosynthesised AgNPs. The MBC values obtained for AgNP samples against *S. aureus* were 400 µg/mL from EG5, and 800 µg/mL from EG6, EG8, and EC2 AgNPs. The MBC values obtained for AgNP samples against *P. aeruginosa* were 100 µg/mL for all AgNP samples. The biosynthesised AgNPs exhibited antibacterial activity against both Gram-positive and Gram-negative bacteria; however, the effect was stronger against *S. aureus* than *P. aeruginosa*. This was evidenced by the lower MIC and MBC values observed for *S. aureus*. In contrast, *P. aeruginosa* required higher concentrations of AgNPs to achieve similar antibacterial effects. The greater susceptibility of *S. aureus* may be attributed to differences in cell wall structure [64]. As a Gram-positive bacterium, *S. aureus* possesses a thick peptidoglycan layer but lacks the outer membrane present in Gram-negative bacteria [65]. Conversely, *P. aeruginosa* has an additional outer membrane rich in lipopolysaccharides that acts as a protective barrier, reducing nanoparticle penetration and contributing to its intrinsic resistance to antimicrobial agents [66].

#### 2.4.2. Zone of Inhibition (ZOI) Measurements

**Figure 8:**
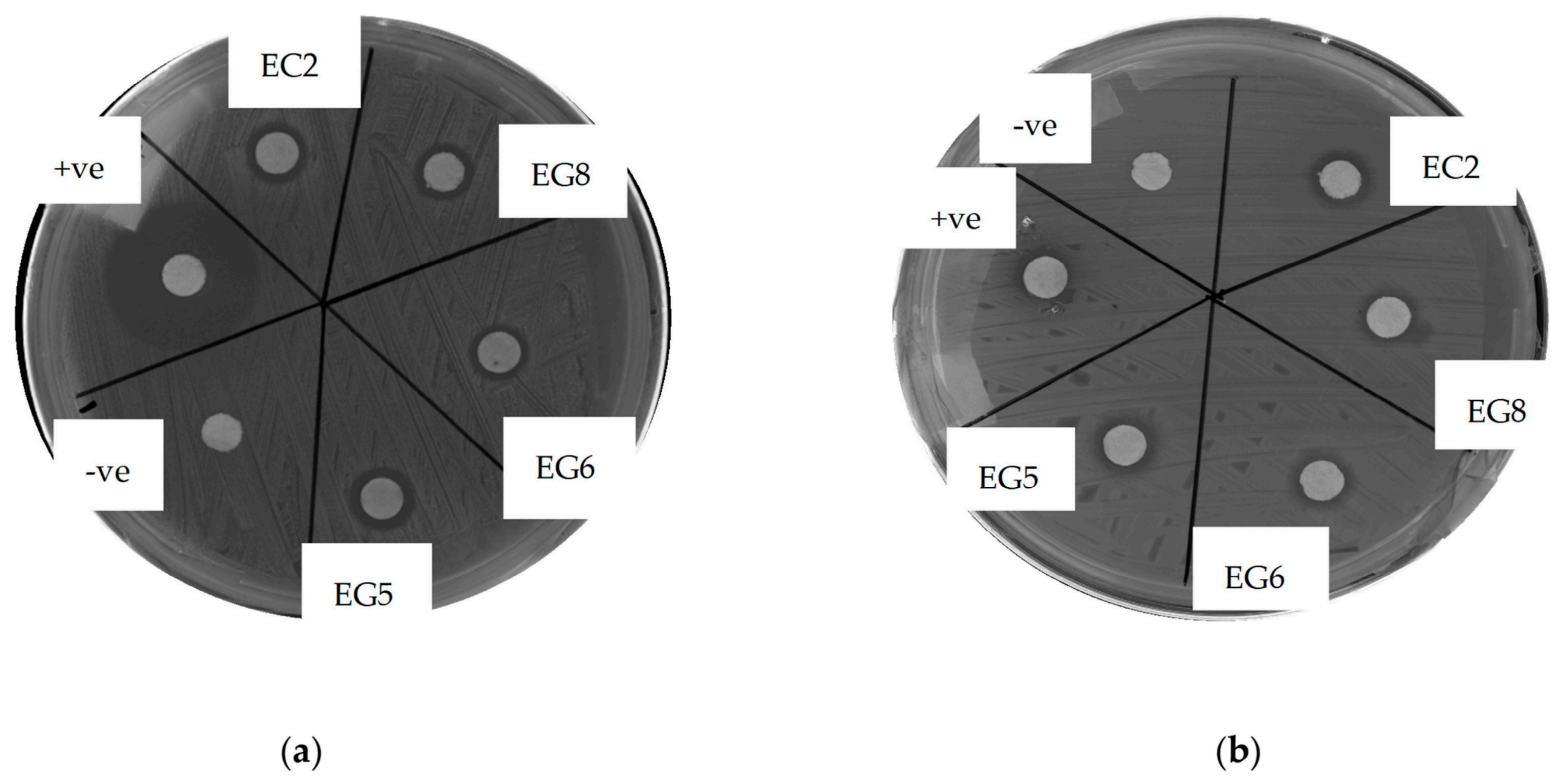
ZOI measurements for AgNPs synthesised from the four fungal isolates tested against (**a**) *S. aureus* and (**b**) *P. aeruginosa*. Ampicillin (20 µg/mL) served as the positive control, while distilled water was used as the negative control.

Following a 24 h incubation period, ZOI was detected surrounding discs enriched with AgNPs and ampicillin. Figure 8 and Table 4 clearly show the strong antibacterial activity of the tested AgNPs against *S. aureus* and *P. aeruginosa* bacteria. The maximum ZOI was observed from EG 5 AgNPs against *S. aureus* (14.6 ± 0.1 mm), followed by EG 6 AgNPs (10.6 ± 0.15 mm), EG 8 AgNPs (12 ± 0.11 mm), and EC 2 AgNPs (12.3 ± 0.05 mm). Against *P. aeruginosa*, EG 5 AgNPs exhibited the highest ZOI (10.6 ± 0.05 mm), followed by EG 6 AgNPs (8.3 ± 0.28 mm), EG 8 AgNPs (9 ± 0.4 mm), and EC 2 AgNPs (10 ± 0.1 mm). The ZOIs around ampicillin discs were 22 ± 0.25 mm and 10 ± 0.15 mm against *S. aureus* and *P. aeruginosa*, respectively. Conversely, no inhibition zones were detected around the distilled water discs.

#### 2.4.3. Growth Inhibition Study

**Figure 9:**
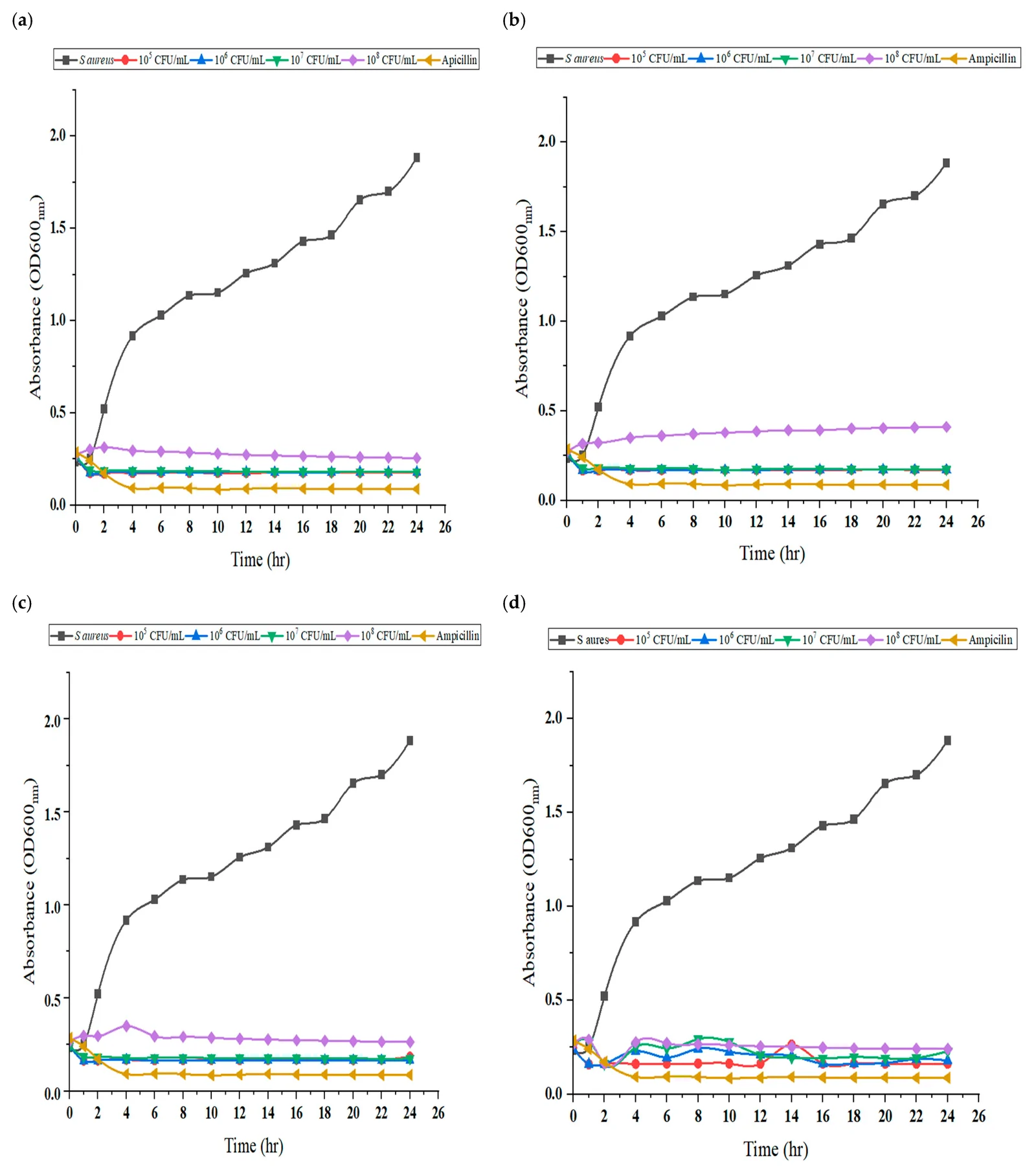
Growth inhibition effect of AgNPs synthesised from (**a**) EG5, (**b**) EG6, (**c**) EG8, and (**d**) EC2, showing the concentration-dependent suppression of *S. aureus* over the incubation period.

**Figure 10:**
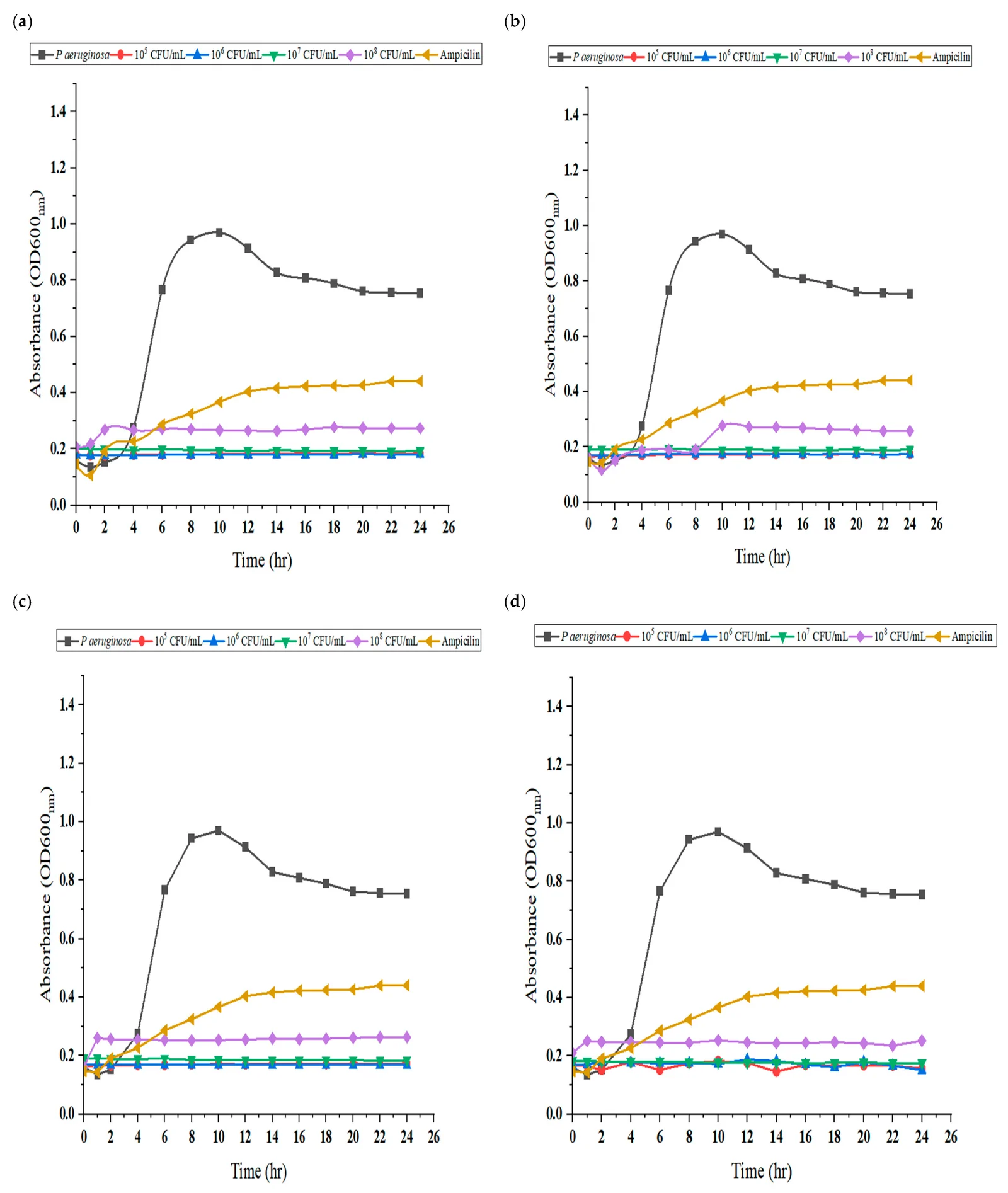
Growth inhibition effect of AgNPs synthesised from (**a**) EG5, (**b**) EG6, (**c**) EG8, and (**d**) EC2, showing the concentration-dependent suppression of *P. aeruginosa* over the incubation period.

Growth kinetic analysis is used to evaluate the time-dependent antibacterial activity of AgNPs and to monitor bacterial proliferation [67]. As shown in Figure 9, the growth of *S. aureus* at all tested inoculum levels (10^8^, 10^7^, 10^6^, and 10^5^ CFU/mL) declined markedly as incubation time increased. The figure also demonstrates that *S. aureus* OD was reduced by all AgNP samples within the first 2 h of incubation, after which bacterial levels remained stable throughout the 24 h. In contrast, untreated bacteria continued to grow without signs of inhibition. Similarly, Figure 10 shows that *P. aeruginosa* exhibited a noticeable reduction in OD_600nm_ beginning at 4 h of incubation in the presence of AgNPs. In contrast, the control culture continued to grow over time.

Bacterial growth kinetics typically progress through lag, exponential, and stabilisation phases; however, the decline phase was not observed in this study because both viable and non-viable cells contribute to absorbance at OD_600nm_. As incubation progressed, the exponential phase was markedly reduced, and the stabilisation phase was reached much earlier, within 0–4 h for *S. aureus* and 0–5 h for *P. aeruginosa* in the presence of AgNPs. These findings indicate that AgNPs exert a concentration- and time-dependent inhibitory effect on bacterial growth. The attachment of AgNPs to the cell membrane induces structural alterations, including cytoplasmic condensation and membrane detachment, ultimately compromising cell wall integrity [68]. Beyond electrostatic interactions, AgNPs also bind to sulphur-containing biomolecules, causing irreversible modifications that further disrupt the bacterial cell wall [69]. Treatment with 20 µg/mL ampicillin significantly reduced the growth of *P. aeruginosa* compared with the untreated control. Although the optical density increased gradually during incubation, the final OD values remained markedly lower than those of the control culture, indicating partial growth inhibition. The inability of ampicillin to completely suppress growth is consistent with the intrinsic resistance mechanisms of *P. aeruginosa*, including low outer-membrane permeability and β-lactamase production [70].

## 3. Materials and Methods

### 3.1. Isolation of Endophytic Fungi

The *Eucalyptus citerodora* and *Eucalyptus globulus* plants were collected from the Kuranga Native Nursery (Mount Evelyn, Victoria, Australia). Healthy plant leaves were used for the isolation of endophytic fungi according to the standard procedure adopted from Bagur et al. [71], with slight modifications. The leaves were surface-sterilised by rinsing under running tap water (room temperature) for 1 h, and then cut into 5 × 5 cm segments and immersed in 70% ethanol for 1 min, followed by sterile distilled water for another 1 min, and this was repeated twice.

The small leaf fragments were then placed on potato dextrose agar (PDA) plates and incubated at 25 °C for 2 weeks, until the hyphal filaments of endophytic fungi developed. Daily observations of growth were conducted, and individual hyphal tips were promptly selected and transferred to a new PDA medium until pure fungal colonies were detected.

The fungal hyphae were recognised by the naked eye, and the pure cultures were obtained by repetitive subculturing. Morphological identification of pure isolates of endophytic fungi was conducted under a light microscope based on colony characteristics and microscopic features.

Genomic DNA from four endophytic fungal isolates was extracted from freshly harvested mycelia following the protocol of Yang et al. [72]. The internal transcribed spacer (ITS) region of the 18S rRNA gene was amplified using the fungal-specific primers ITS1 (5′-TCGTAGTGACCTGCGGAGGGA-3′) and ITS4 (5′-CTGATCCGAGGTCAAAGTTGAAA-3′). PCR amplification was performed under standard cycling conditions, and the resulting amplicons were verified by agarose gel electrophoresis before being purified for sequencing. Sequencing results were analysed using BLAST option to determine the closest matches based on maximum identity scores. Multiple sequence alignments were generated, and distance matrices were calculated to assess genetic relatedness. Phylogenetic trees were constructed using the maximum likelihood with bootstrap analysis to evaluate branch reliability, employing MEGA11 software.

### 3.2. Preparation of Extracellular Extract

The extracellular extraction was performed according to Murillo et al. [73] with minor modifications. The endophytic fungal samples were mass-produced in potato dextrose broth (PDB) flasks, and incubated in an orbital shaker at 25 ± 2 °C and 150 rpm for 14 days. The fungal mycelia were harvested and washed with sterilised Milli-Q water three times to avoid contamination of medium components. Approximately 10 g of wet weight of fungal biomass was added to 100 mL of Milli-Q water and incubated in an orbital shaker under the abovementioned conditions. After 72 h, the second filtration was performed, and the extracellular extract was used as a metal-ion reducer to form AgNPs.

### 3.3. Biosynthesis of AgNPs

The extracellular extract was mixed with 1 mM AgNO_3_ in a 1:1 (*v*/*v*) ratio and incubated at 30 °C with shaking at 200 rpm under dark conditions to prevent light-induced reactions. After 72 h, the reaction mixture was examined for a colour change to brown or dark brown, indicating the successful formation of AgNPs. This procedure was adapted from Gezaf et al. [74], with minor modifications. The biosynthesised AgNPs were separated from the reaction mixture by centrifugation at 15,000 rpm for 30 min. The resulting pellet was washed twice with Milli-Q water to remove residual fungal metabolites and unreacted components. The purified AgNPs were subsequently freeze-dried to obtain a dry AgNP powder, which was stored at 4–8 °C until further use. Prior to antibacterial assays, the freeze-dried AgNP powder was dissolved in Milli-Q water, ultrasonicated for 30 min and diluted to the desired concentrations for experimental applications.

### 3.4. Characteristics of Biosynthesised AgNPs

#### 3.4.1. UV–Visible Spectroscopy (UV-Vis)

UV-Vis measurement was performed. The UV-Vis absorbance of the formed solution was assessed using a Cary UV-Vis-NIR spectrometer (Agilent Technologies, Santa Clara, CA, USA) within the wavelength range of 250 to 750 nm, scanned at a resolution of 1 nm.

#### 3.4.2. Dynamic Light Scattering (DLS)

Measurements of average particle size distribution, polydispersity index (PDI), and zeta potential of a colloidal solution containing AgNPs were performed using a Thermo Scientific™ Nicolet iS™5 Malvern Zeta Potential Particle Sizer (Thermo Fisher Scientific Inc., Madison, WI, USA).

#### 3.4.3. Fourier-Transform Infrared Spectroscopy (FTIR)

The FTIR analysis was conducted using a Thermo Scientific™ Nicolet iS™50 FTIR spectrophotometer (Thermo Fisher Scientific Inc., Madison, WI, USA) within the range of 500–4000 cm^−1^.

#### 3.4.4. Transmission Electron Microscopy (TEM)

The morphology and size of the AgNPs were evaluated using a TEM (JEOL JEM 1010) Electron Microscopy (JEOL Ltd., Tokyo, Japan), conducted at RMIT University, Melbourne, Australia. Each AgNP was prepared at a 500 µg/mL concentration, and a 5 µL drop was cast on holey carbon grids until fully dried. The grids were used to detect the morphological characteristics and distribution of AgNPs. TEM images were analysed using Fiji: ImageJ software, and the diameters of 150 individual nanoparticles were manually measured to calculate the mean particle size and standard deviation.

#### 3.4.5. X-Ray Photoelectron Spectroscopy (XPS)

XPS analysis was employed to determine the elemental composition and surface chemical characteristics of the biosynthesised AgNPs using a Kratos Nova X-ray Photoelectron Spectrometer (Kratos Analytical, Manchester, UK). A suspension of freeze-dried AgNPs was prepared at a concentration of 25 µg/mL in Milli-Q water and was sonicated for 30 min to minimise nanoparticle aggregation. Subsequently, 20 µL of the suspension was deposited onto a sterile silicon wafer substrate (approximately 10 mm × 10 mm) using the drop-casting method. The samples were allowed to air-dry under sterile conditions at room temperature to ensure complete solvent evaporation and the formation of a thin, homogeneous AgNPs film on the wafer surface. After drying, the samples were carefully mounted onto the sample holder and analysed by XPS. Survey spectra and elemental composition analyses were processed using CasaXPS software version 2.1.0.1.

### 3.5. Bacterial Strains

*Staphylococcus aureus* (ATCC12600) and *Pseudomonas aeruginosa* (ATCC10145) strains were obtained from Swinburne University of Technology, Australia, Department of Chemistry and Biotechnology. Fresh bacterial cultures were prepared in nutrient broth (NB) and incubated at 37 °C for 24 h.

### 3.6. Minimum Inhibitory Concentration and Minimum Bacterial Concentration

MIC was determined using the broth microdilution approach in sterile 96-well microplates. AgNPs suspensions were prepared at predetermined concentrations (60, 80, 100, 400, 800, and 1000 µg/mL) and dispersed in sterile Milli-Q water, and then ultrasonicated for 30 min prior to use to ensure uniform distribution. Each well contained a final volume of 200 µL, consisting of bacterial inoculum and AgNPs suspension at the desired concentration. The bacterial inoculum was adjusted to approximately 0.5 McFarland’s standard. All experiments were performed in triplicate. The MIC was defined as the lowest AgNPs concentration that completely inhibited visible bacterial growth compared with the growth control. Growth inhibition was assessed visually.

After the MIC, the minimum bactericidal concentration (MBC) of AgNPs was determined for each bacterial pathogen. Aliquots (50 µL) from wells showing no visible bacterial growth were streaked onto Mueller–Hinton agar (MHA) plates and incubated at 37 °C for 24 h. Bactericidal activity was assessed by examining the plates for bacterial colony formation after incubation. The MBC was defined as the lowest AgNPs concentration at which no visible bacterial colonies were observed on the agar plates. The MBC values were based on the presence or absence of colony growth. The MIC and MBC procedure was followed by the method reported by Sheng et al. [75]. Aliquots from the MIC assays showing no visible bacterial growth were streaked onto fresh agar plates and incubated under the same conditions. The absence of bacterial growth on the subcultured plates indicated that the bacteria had been killed rather than merely inhibited.

### 3.7. Kirby–Bauer Disc Diffusion Assay

The antibacterial activity of the biosynthesised AgNPs was evaluated using the disc diffusion assay [76]. The surface of Mueller–Hinton agar (MHA) plates was uniformly inoculated with the bacterial suspension, and sterile paper discs were impregnated with 10 µL of AgNPs at their respective MIC concentrations and placed onto the inoculated agar surface. Sterile distilled water (10 µL) was used as the negative control, while discs loaded with 10 µL of ampicillin solution (20 µg/mL) served as the positive control. The plates were incubated at 37 °C for 24 h, after which the antibacterial activity was assessed by measuring the diameter of the zone of inhibition (ZOI) surrounding each disc using a ruler.

### 3.8. Growth Inhibition Assay

The growth–kill curve was created according to the protocol by Haque et al. [67] using a 96-well plate. Growth curve analyses were performed in the presence of biosynthesised AgNPs at concentrations corresponding to the MIC values previously determined for each bacterial strain. Bacterial concentration was adjusted from an overnight culture of *S. aureus* and *P. aeruginosa*, equivalent to 10^5^, 10^6^, 10^7^, 10^8^ CFU/ml; 10^8^ CFU/ml bacteria alone were used as a negative control, and a mix of bacteria with ampicillin 20 µg/mL was used as a positive control. The final readings were obtained from a microplate reader after 24 h of incubation at 37 °C in a series of time intervals for every hour and recorded based on OD_600nm_. The corrected absorbance values used for MIC determination were calculated by subtracting the absorbance of the corresponding AgNP blank from the absorbance of the test well to eliminate any background absorbance arising from the AgNPs, which may contribute to the optical density measurements independently of bacterial growth.

The corrected absorbance was calculated using Equation (2):

Corrected Absorbance = *A*_sample_ − *A*_βlank_ (2)

where A_sample_ represents the absorbance of the well containing bacterial growth, growth medium, and AgNPs, and A_βlank_ represents the absorbance of the corresponding well containing the same concentration of AgNPs and growth medium but without bacterial growth.

### 3.9. Statistical Analysis

All experiments were performed in triplicate, and the results are expressed as mean ± standard deviation (SD). The antibacterial activity data, including ZOI and MIC values, as well as the average particle size obtained from TEM analysis, were analysed using Microsoft Excel. For TEM analysis, the average particle size was calculated from multiple particles for each sample. The results are reported as mean particle diameter ± SD.

## 4. Conclusions

In this study, AgNPs were successfully biosynthesised using extracellular extracts from four endophytic fungal isolates obtained from Australian native plants. UV–Vis spectroscopy confirmed AgNP formation through characteristic surface plasmon resonance (SPR) bands in the 400–500 nm range. DLS and TEM analyses revealed predominantly spherical NPs with different size distributions, consistent with effective antimicrobial AgNPs reported in the literature. The observed antibacterial activity of the biosynthesised AgNPs correlated with their nanoscale size, as smaller, well-dispersed particles provided a higher surface area for interaction with bacterial cell membranes, thereby enhancing antimicrobial efficacy.

Notably, variations in particle size distribution, surface charge, and antibacterial effectiveness among the different fungal-derived AgNPs suggest that the biochemical composition of the extracellular extracts plays a critical role in nanoparticle synthesis. FTIR analysis indicated the involvement of fungal biomolecules such as proteins, polysaccharides, and phenolic compounds, which acted as both reducing and stabilising agents. Differences in the type and concentration of these biomolecules likely influenced the biosynthesis process, resulting in distinct physical properties of the AgNPs, including size heterogeneity. These compositional differences in the fungal extracts therefore contributed to variations in NP characteristics and, consequently, their antibacterial performance. Overall, this study demonstrates that endophytic fungus-mediated synthesis offers a green and promising approach for producing biologically active AgNPs. The ability to modulate NP properties through fungal extract composition highlights the potential of these systems as a promising source of effective antibacterial nanomaterials.

## Figures and Tables

**Table 1:** Average size, PDI, and zeta potential of AgNP samples from EG5, EG6, EG8, and EC2 endophytic fungal samples.

AgNP Sample	Avg. Size	PDI	Zeta Potential
EG5	80	0.19	−19.3
EG6	158	0.41	−31.7
EG8	121	0.36	−18.1
EC2	104	0.52	−21.9

**Table 2:** MIC screening of AgNPs against *S. aureus*.

AgNPs	AgNP Concentration (µg/mL)						Control	
	60	80	100	400	800	1000	Bacterial Growth	Ampicillin 20 µg/mL
EG5	+	+	−	−	−	−	+	−
EG6	+	+	+	−	−	−	+	−
EG8	+	+	+	−	−	−	+	−
EC2	+	+	+	−			+	−

Positive (+): turbidity; negative (−): no turbidity.

**Table 3:** MIC screening of AgNPs against *P. aeruginosa*.

AgNPs	AgNP Concentration (µg/mL)						Control	
	60	80	100	400	800	1000	Bacterial Growth	Ampicillin 20 µg/mL
EG5	+	+	+	+	−	−	+	−
EG6	+	+	+	+	+	−	+	−
EG8	+	+	+	+	+	−	+	−
EC2	+	+	+	+	+	−	+	−

Positive (+): turbidity; negative (−): no turbidity.

**Table 4:** Average zone of inhibition of four AgNPs against *S. aureus* and *P. aeruginosa*.

Bacterial Pathogen	Zone of Inhibition (mm)					
	Ampicillin 20 µg/mL	Distilled Water	EG 5 AgNPs	EG 6 AgNPs	EG 8 AgNPs	EC 2 AgNPs
*S. aureus*	22 ± 0.25	0	14.6 ± 0.1	10.6 ± 0.15	12 ± 0.11	12.3 ± 0.05
*P. aeruginosa*	10 ± 0.15	0	10.6 ± 0.05	8.3 ± 0.28	9 ± 0.4	10 ± 0.1

## Data Availability

Data supporting this study are available from the corresponding author upon reasonable request.

## Supplementary Materials

The following supporting information can be downloaded at https://www.mdpi.com/article/10.3390/molecules31193505/s1, Figure S1: The phylogenetic relationships inferred using the maximum likelihood method for endophytic fungal samples EG5, EG6, EG8, and EC2 with *Fusarium venenatum* included as the outgroup. Bootstrap analysis was performed with 1000 replications to assess branch reliability. Scale bar = 0.09 substitutions per nucleotide position. Figure S2: Graphs a–d show the particle size distribution profiles for AgNPs synthesised from (a) EG5, (b) EG6, (c) EG8, and (d) EC2.

## Abbreviations

The following abbreviations are used in this manuscript:

AgNPs	Silver Nanoparticles
NPs	Nanoparticles
UV-vis	UV–visible spectroscopy
TEM	Transmission electron microscopy
FTIR	Fourier-transform infrared spectroscopy
*S. aureus*	*Staphylococcus aureus*
*P. aeruginosa*	*Pseudomonas aeruginosa*
potato dextrose agar	PDA
MIC	minimum inhibitory concentration
MBC	minimum bactericidal concentration
PDI	polydispersity index
MHA	Mueller-Hinton Agar
