# Supplementary material for: Screening Endophytic Fungi of Australian Indigenous Plants for Biosynthesis of Silver Nanoparticles and Evaluation of Their Antibacterial Properties
Source: Molecules. 2026 Oct 1;31(19):3505. doi: 10.3390/molecules31193505 (PMC13666900; doi:10.3390/molecules31193505)

Supplementary Information

Figure S1: The phylogenetic relationships inferred using the maximum likelihood method for endophytic fungal samples EG5, EG6, EG8, and EC2 with *Fusarium venenatum* included as the outgroup. Bootstrap analysis was performed with 1000 replications to assess branch reliability. Scale bar = 0.09 substitutions per nucleotide position.

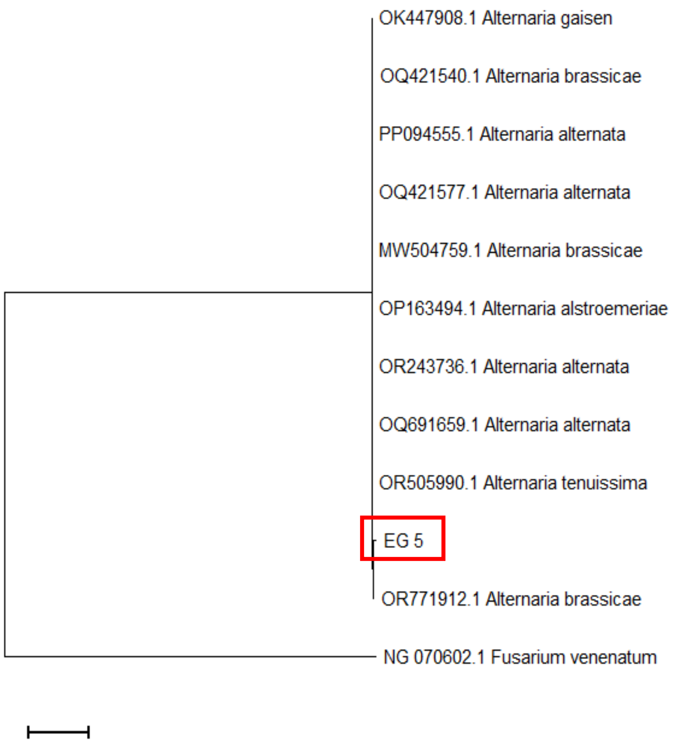

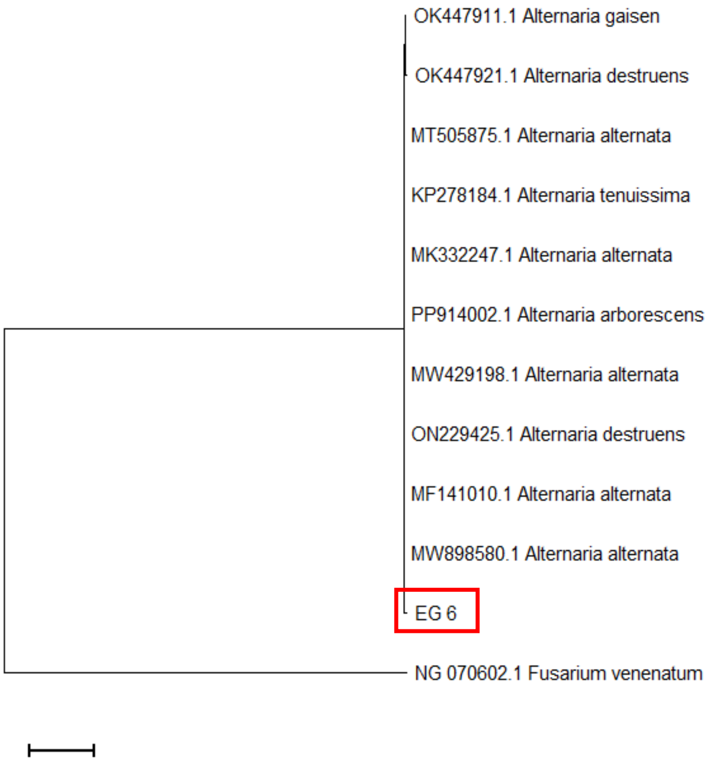

Internal

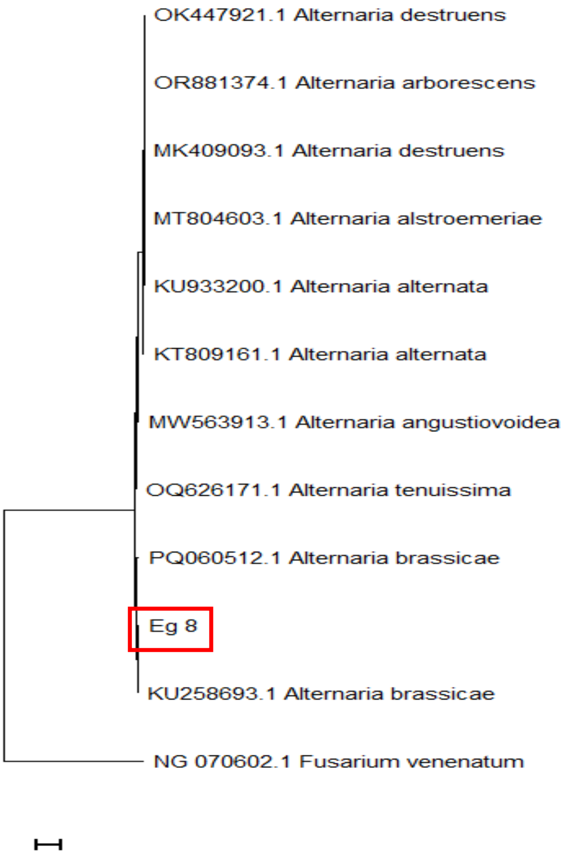
OK447921.1 Alternaria destruens
OR881374.1 Alternaria arborescens
MK409093.1 Alternaria destruens
MT804603.1 Alternaria alstroemeriae
KU933200.1 Alternaria alternata
KT809161.1 Alternaria alternata
MW563913.1 Alternaria angustiovoidea
OQ626171.1 Alternaria tenuissima
PQ060512.1 Alternaria brassicae
Eg 8
KU258693.1 Alternaria brassicae
NG 070602.1 Fusarium venenatum

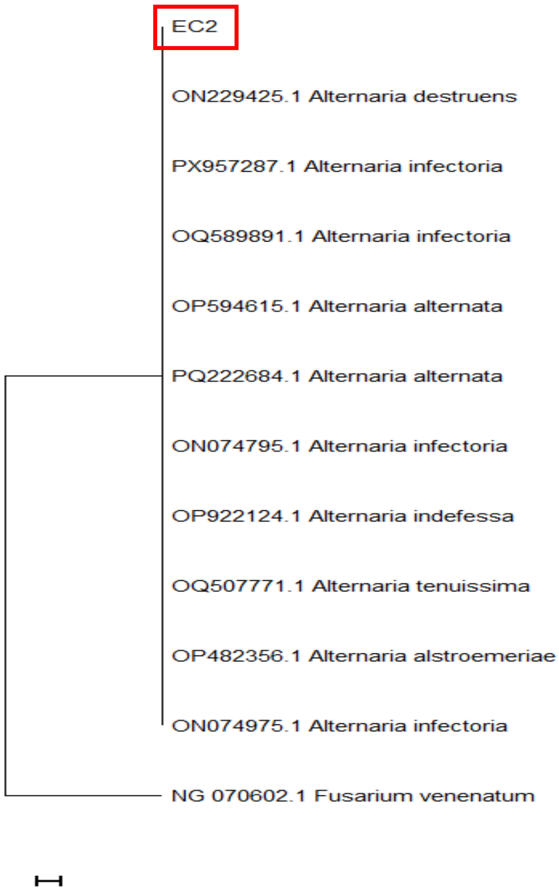
EC2
ON229425.1 Alternaria destruens
PX957287.1 Alternaria infectoria
OQ589891.1 Alternaria infectoria
OP594615.1 Alternaria alternata
PQ222684.1 Alternaria alternata
ON074795.1 Alternaria infectoria
OP922124.1 Alternaria indefessa
OQ507771.1 Alternaria tenuissima
OP482356.1 Alternaria alstroemeriae
ON074975.1 Alternaria infectoria
NG 070602.1 Fusarium venenatum

Internal

Figure S2: Graphs a–d show the particle size distribution profiles for AgNPs synthesised from (a) EG5, (b) EG6, (c) EG8, and (d) EC2.

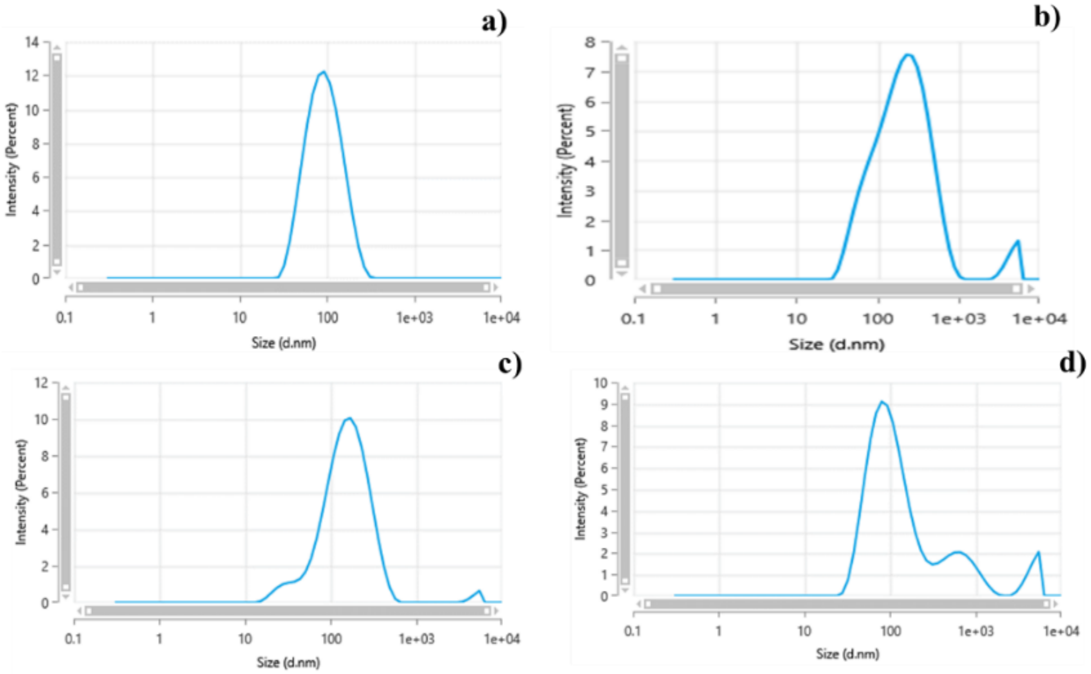

Supplement: Supplementary file 1 [file molecules-31-03505-s001.zip › molecules-4556259-supplementary.pdf]
